# A Rapid Sound-Action Association Effect in Human Insular Cortex

**DOI:** 10.1371/journal.pone.0000259

**Published:** 2007-02-28

**Authors:** Isabella Mutschler, Andreas Schulze-Bonhage, Volkmar Glauche, Evariste Demandt, Oliver Speck, Tonio Ball

**Affiliations:** 1 Epilepsy Center, University Hospital Freiburg, Freiburg, Germany; 2 Heidelberg Academy of Science and Humanities, Heidelberg, Germany; 3 Freiburg Brain Imaging, University Hospital Freiburg, Freiburg, Germany; 4 Bernstein Center for Computational Neuroscience Freiburg, Freiburg, Germany; 5 Neurobiology and Animal Physiology, Institute for Biology I, University of Freiburg, Freiburg, Germany; 6 Department of Diagnostic Radiology, Medical Physics, University Hospital Freiburg, Freiburg, Germany; University of St. Andrews, United Kingdom

## Abstract

**Background:**

Learning to play a musical piece is a prime example of complex sensorimotor learning in humans. Recent studies using electroencephalography (EEG) and transcranial magnetic stimulation (TMS) indicate that passive listening to melodies previously rehearsed by subjects on a musical instrument evokes differential brain activation as compared with unrehearsed melodies. These changes were already evident after 20–30 minutes of training. The exact brain regions involved in these differential brain responses have not yet been delineated.

**Methodology/Principal Finding:**

Using functional MRI (fMRI), we investigated subjects who passively listened to simple piano melodies from two conditions: In the ‘actively learned melodies’ condition subjects learned to play a piece on the piano during a short training session of a maximum of 30 minutes before the fMRI experiment, and in the ‘passively learned melodies’ condition subjects listened passively to and were thus familiarized with the piece. We found increased fMRI responses to actively compared with passively learned melodies in the left anterior insula, extending to the left fronto-opercular cortex. The area of significant activation overlapped the insular sensorimotor hand area as determined by our meta-analysis of previous functional imaging studies.

**Conclusions/Significance:**

Our results provide evidence for differential brain responses to action-related sounds after short periods of learning in the human insular cortex. As the hand sensorimotor area of the insular cortex appears to be involved in these responses, re-activation of movement representations stored in the insular sensorimotor cortex may have contributed to the observed effect. The insular cortex may therefore play a role in the initial learning phase of action-perception associations.

## Introduction

Interest in the functional linkage between the auditory and motor systems has increased in the last few years. Auditory-motor integration has been investigated in musical performance and training, and in connection with every-day action-related sounds [1], [2]. For instance, silent tapping of a violin concerto has been found to be associated with greater activation of the primary auditory cortex in professional musicians than in non-musicians [2], suggesting a functional link from the motor to the auditory system that is sensitive to training. Conversely, there is also evidence for a functional link from the auditory system to the motor system: For instance, passive listening to action-related sounds such as the sound of ripping a sheet of paper activates a left-hemispheric temporo-parieto-premotor circuit that includes the supplementary motor area (SMA) and Broca's area. This finding has been interpreted in favor of the existence of an ‘auditory mirror neuron system’ in humans [1]. The sound-action associations investigated in this study were evolutionarily novel, and it has therefore been argued that the observed ‘mirror’ activations reflect *learned* associations between novel actions and their sounds that were established over a long time before the actual experiments [1].

Recent studies have addressed the question of how processing of auditory stimuli changes following acquisition of sound-action associations. Bangert and colleagues [3] investigated cortical activation patterns using DC-EEG-recordings obtained in subjects who listened passively to a musical piece before and after learning to play the piece on the piano. The recordings showed wide-spread EEG potential changes over fronto-parietal areas that were already present after the first training session. The authors interpreted their findings as an indication of auditory sensorimotor co-activation. Using a similar learning paradigm, we investigated non-musicians who were instructed to learn simple melodies on a piano with their right hand [4]. Single pulse-induced motor evoked potentials (MEPs) obtained by stimulation above the left hemisphere were recorded from the first dorsal interosseus muscle of the right hand prior to and after the learning procedure while subjects listened passively to the learned melodies, unknown melodies, and to white noise. We found a trend toward greater amplitudes of MEPs during the exposure to learned melodies than during exposure to novel melodies or noise. Also using TMS, D'Ausilio and co-workers [5] compared motor cortical excitability during passive listening to previously rehearsed piano melodies with passive listening to control melodies. This study also demonstrated motor cortical excitability changes for the rehearsed compared to the unrehearsed piece.

The techniques used in these previous studies allow however only a limited assessment of the exact cortical networks that generate the observed effects. EEG signals measured on the scalp surface as in the study of Bangert and colleagues [3] do not directly indicate the exact number and position of the underlying generators. This is due to the blurring effect of the interposed volume conductor and the ambiguity of the resulting electromagnetic inverse problem [for a recent review see [6]]. TMS procedures as used in the studies cited above [4], [5] evaluate neuronal excitability only in the few square cm of cortex that are directly targeted by the stimulation coil [7], [8]. The results obtained do not indicate which subcortical and/or up-stream cortical areas shape the observed motor cortical excitability changes.

In view of the preceding considerations, the aim of the present study was to apply an imaging method with high localization accuracy in order to determine the brain areas responsible for the differential brain responses to rehearsed musical pieces. Using functional magnetic resonance imaging (fMRI), we investigated healthy subjects during the presentation of actively and passively learned piano melodies. Our results provide the first evidence that, compared with passively learned melodies, actively learned melodies evoke increased fMRI responses already after training periods of 30 minutes or less in the left anterior insula overlapping the insular sensorimotor hand representation area.

## Methods

### Subjects

Ten subjects without any previous experience in playing the piano and without any other professional music education took part in this study (5 females, 5 male, mean age = 27.1 years, age range = 20–41 years) after giving written informed consent. The study was approved by the ethics committee of the University of Freiburg, Germany. All participants were healthy, with no past history of psychiatric or neurological disease or hearing problems. Subjects were right-handed according to the Edinburgh Handedness Inventory [9]: mean = 89.3%, range = 67–100%.

### Stimuli and Procedure

**Figure 1:**
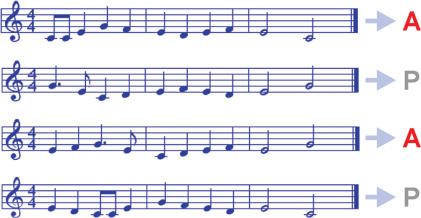
Active/passive melody learning task. Subjects learned to play two unknown melodies on a piano (actively learned melodies, marked ‘A’) and were passively familiarized with two other melodies (passively learned melodies, marked ‘P’). The assignment to the two learning conditions was balanced across subjects.

Subjects learned with their right hand to play two simple melodies on a piano (Fig. 1). Five subjects learned melodies 1 and 4 and five subjects learned melodies 2 and 3 by rehearsing the melodies, that is, by alternating between listening and playing (‘actively learned melodies’). Subjects were blindfolded to ensure that learning relied on auditory feedback. Additionally, subjects were familiarized with two other melodies without playing them (‘passively learned melodies’). The experimental set-up consisted of a Yamaha Disklavier connected to a computer. The melodies were presented using Cubase VST/32 R.5 (Steinberg) software. The learning criterion for each melody was to be able to play the tune twice without making a mistake. After fulfilling the learning criterion for the first melody, subjects learned to play the second melody. In addition, subjects learned the remaining two melodies passively by listening to each melody 10 times (‘passively learned melodies’). To ensure that actively and passively learned melodies had the same degree of familiarity, we conducted a performance test for familiarity: the four melodies were presented in random order and slight variations were built into 7 of the 15 repetitions of each melody. This was achieved by shifting the pitch of a single note in the first, the second or the third bar to either a note higher or a note lower than in the original version. Subjects were required to indicate with a computer mouse whether the melodies corresponded with the original version of the melody or not. No feedback was given as to the correctness of the subjects' answers. The stimuli of the performance test for familiarity were presented to the subjects using in-house developed software and the sound module of the Yamaha Disklavier with headphones (Hanumpa, Digital Pro-H700, Tempest). Together, the learning phase and the detection task lasted approximately 60 minutes. After a time interval of 20 minutes, an fMRI experiment was conducted, in which the actively and passively learned piano melodies (wave files) were presented in random order via magnetic resonance compatible headphones (Nordic Neuro Lab Norway) to the subjects, using an in-house developed presentation software. Subjects viewed a fixation cross during the experiment and were instructed to listen attentively to the music and to avoid any overt movement. Each stimulus began with a written instruction presented on the screen (‘music starts’). Each melody was presented 10 times, and lasted 9 seconds. After each melody presentation a period of 15 sec. without music presentation followed. Functional and structural measurements lasted 25 minutes.

After the fMRI measurement subjects self-assessed valence (ranging from 0 = unpleasant to 6 = pleasant) and arousal (ranging from 0 = calming to 6 = arousing) of the actively and passively learned melodies. In addition, participants rated their tendency to execute hand movement, to imagine hand movement (both ranging from 0 = none, 1 = rarely, 2 = frequently, to 3 = all the time) and mode of movement imagination (ranging from 0 = purely visual, 3 = visual and kinaesthetic to 6 = purely kinaesthetic). Lifetime musical education was assessed using a questionnaire translated and modified from Litle and Zuckerman [10] to ensure that subjects had not played the piano before the experiment.

An analogous experimental procedure has been successfully used in a previous pilot study using transcranial magnetic stimulation (TMS) [4]. Preliminary results of the present fMRI study have previously been presented in abstract from [11].

### Magnetic Resonance Imaging

Functional and structural images were acquired on a 3T scanner (Siemens Magnetom Trio, Erlangen, Germany). Structural T1-weighted images with 1 mm isotropic resolution were obtained using the MPRAGE sequence. Functional images were acquired using a multislice gradient echo planar imaging method (EPI). Within 44 sagittal slices the entire brain was included (TR 3000 ms, TE 30 ms, 90° flip angle, 3 mm isotropic resolution). Phase encoded direction was anterior-posterior. The sagittal slice orientation resulted in significantly lower acoustic noise generated by the imaging gradients, enabling a better auditory stimulus perception. In addition, this orientation in combination with the thin slice thickness reduced the signal loss to give more reliable detection of activation.

Accurate registration of the functional and structural images was ensured by correcting the EPI data for geometric distortions [12]. The distortion field was derived from the local point spread function in each voxel determined in a one minute reference scan. Prior to distortion correction, the data was motion corrected by registration to the position of the reference scan. Motion and distortion correction were performed online during the reconstruction process.

### MRI Data analysis

The data of the subjects' ratings and the detection rate of melody variations in the performance test for familiarity were analyzed with SPSS 13.0 by calculating non-parametric tests (sign test). For spatial pre-processing and statistical analyses of the functional MR data, the statistical parametric mapping software package (SPM5, Wellcome Department of Cognitive Neurology, London, UK) was used. All functional images were realigned to the mean EPI volume, normalized into standard stereotaxic space (MNI template provided by the Montreal Neurological Institute), and smoothed using an 8 mm full-width-at-half-maximum (FWHM) Gaussian kernel. The two music conditions were modeled with a box-car function convolved with a canonical hemodynamic response function in the General Linear Model of SPM5. A high-pass filter with a cut-off of 1/128 Hz was applied to the voxels' time series. For the statistical analysis, t-contrast images of actively learned>passively learned melody presentation were calculated at the individual level and were used for the random effects second-level analyses (one sample T-test). Additionally, we calculated group level regression analyses with the following parameters as regressors: (1) the subjects' rating of their tendency to movement imagination, (2) the total number of learning trials needed by each subject to reach the learning criterion for the actively learned melodies, and (3) the difference of correctly identified variations for the actively learned melodies minus for the passively learned melodies (‘familiarity difference’),

For the main contrast of interest, namely listening to actively>passively learned melodies, we report results at p<0.005, k>100, and Z-score>3.0 in the *a priori* regions of interest. As summarized in the introduction, the results of previous studies indicate a sensorimotor co-activation when listening to rehearsed pieces. We therefore defined as our *a priori* region of interests the sensorimotor cortical regions subserving hand motor control (including the primary, premotor, supplementary motor, and cingulate motor areas, as well as the sensorimotor region of the insula and fronto-opercular cortex [13]). There were no activations in the correlation analyses at this threshold. In addition, we show whole brain results at a lower statistical threshold of p<0.05, k> = 50, and Z-score>2.25. To assign peaks-activations to anatomical areas, the SPM Anatomy Toolbox [14] was used. For each BOLD signal change peak the corresponding macro-anatomically defined brain region, the probabilities of belonging to the currently available micro-anatomically defined brain areas, and the maximum-probability-map [15] based peak assignment to the probabilistic-anatomical maps were determined.

### Meta analysis of previous insular movement related fMRI activation

To delineate the region of the insular cortex showing consistently hand movement-related fMRI activation, a meta-analysis of previous fMRI studies was carried out. Studies included in the meta-analysis had to fulfill the following criteria: (1) they had to report hand or finger movement-related BOLD signal changes in right-handed healthy adult subjects, (2) coordinates had to be given either in Talairach or in MNI (Montreal Neurological Institute) space. 20 studies meeting these criteria were surveyed [16]–[35]. In total, 42 stereotaxic coordinates (23 on the left, 19 on the right hemisphere) were analyzed. Talairach coordinates were translated to match the MNI space. The reported foci were treated as localization probability distributions centered at the given Y and Z peak coordinates [36]. The probability distribution was modeled by two dimensional Gaussian functions with 8mm FWHM both in the Y and Z direction. Since the included functional imaging data was preprocessed by spatial filtering using Gaussian kernels, this use of Gaussian functions yields an approximation of the volumes underlying the published peak data. The FWHM of 8 mm used for this analysis is within the range of the smoothing filters used in the original studies included in the meta-analysis (from 4 mm to 12 mm). Subsequently an ‘activation likelihood estimate’ (ALE) [36], given by the union of the probabilities associated with the different foci, was calculated for an area comprising the whole Y and Z extent of the insular cortex. The later was determined by manual segmentation from the T1-multi-subject template provided with SPM5.

## Results

Across subjects, the mean number of learning trials needed to reach the learning criterion for both of the actively learned melodies was 20.5 (range 10 to 40 trials). There was no significant difference between the learning trials needed for the first and second melody (p>0.6). First learned melody: mean = 10.4 trials, SD = 5.4 trials; second learned melody: mean = 10.1 trials, SD = 5.86. In six subjects, the number of learning trials was equal for the first and second melody. Two participants needed more and two subjects fewer learning trials for the second as compared with the first melody. There was no significant difference between the number of learning trials for the passively learned melodies (i. e. 10 trials for each melody) and the number of learning trials for the actively learned melodies (sign test, p>0.3). Due to the longer intervals between consecutive melody presentations, the mean total duration of the active learning part of the experiment was longer than the total duration of the passive learning part (15.5 min vs. 8.4 min).

**Figure 2:**
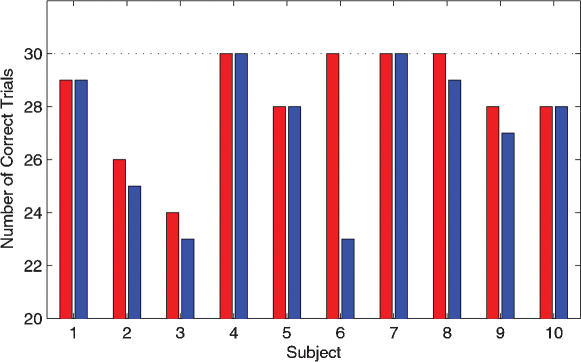
Melody-Variation-detection task. Subjects learned to play two unknown melodies on a piano (actively learned melodies, marked ‘A’) and were passively familiarized with two other melodies (passively learned melodies, marked ‘P’). The assignment to the two learning conditions was balanced across subjects.For each of the ten subjects, the number of correctly identified melodies (either as original or as variation) is given in red for the actively learned melodies and in blue for the passively learned melodies. The total number of trials for each case was 30. The difference between correct trials for the actively and passively learned melodies was used for a correlation analysis to evaluate familiarity effects in the functional data (see below).

The results of the melody-variation detection task are given in Fig. 2. Mean number of correctly identified pieces for the actively learned melodies was 28.3 (SD = 2.0) and for the passively learned melodies 27.2 (SD = 2.66). This tendency to a slightly higher detection rate in the actively learned melodies was not significant at p<0.05 (p = 0.0625, paired sign test).

Mean valence and arousal ratings for the actively learned melodies were 2.6 and 2.4, for the passively learned melodies 2.5 and 2.3. There were no significant differences for arousal and valence ratings between the actively and passively learned melodies at (p>0.9). All subjects rated their tendency to movement execution as ‘none’, consistent with the visual inspection of the subjects by the experimenter. Mean rating of the tendency to movement imagery was 1.0 (corresponding to ‘rarely’); the mode of imagery was rather visual than kinaesthetic (mean 0.87).

**Figure 3:**
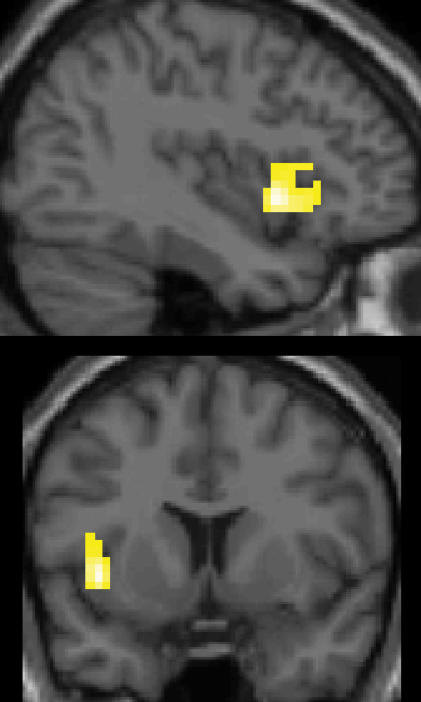
Differential responses to actively and passively learned melodies. Increased BOLD effect in response to the actively learned melodies compared with the passively learned melodies was located in the left anterior insula, extending to the deep fronto-opercular cortex (p<0.005, k>100, Z-score>3.0, slices are at x = −39 and y = 9).

Larger BOLD effect during listening to the actively than during listening to the passively learned melodies was found in the left anterior insula (we report results at p<0.005, k>100, Z-score>3.0 uncorrected for multiple comparisons in the *a priori* regions of interest, e.g. the cortical motor areas, Fig. 3). The activation cluster comprised 147 voxels (cluster level P-value for *a priori* region of interest 0.006) and showed three local maxima with a Z-score>3.0 (see Tab. 1) in the anterior insular cortex. The cluster also extended into the adjacent inferior frontal gyrus/pars opercularis. Additionally, peak locations of BOLD signal changes in the same contrast at a lower threshold (p<0.05, Z>2.25, k>50) are given in Tab. 1. Brain regions with peaks observed at this lower threshold included the middle temporal gyrus and Broca's area.

**Table 1:** For each BOLD signal change, peak MNI-coordinates and z-score are given.

MNI-Coordinates (x/y/z)			Z-Scores	Brain region	Probability	Assigned to
−30	30	6	3.39	Left Insular Cortex (*)	–	–
−39	9	−3	3.29	Left Insular Cortex (*)	–	–
−36	24	0	3.03	Left Insular Cortex (*)	–	–
60	−45	0	3.23	Right Middle Temporal Gyrus	–	–
30	−60	9	3.00	Right Calcarine Gyrus	Area 17: 20%	–
−42	12	9	2.96	Left Inferior Frontal Gyrus (pars opercularis)	Area 44: 30%	–
−27	45	24	2.29	Left Anterior Middle Frontal Gyrus	–	–
21	−6	51	2.95	Left Premotor Cortex	Area 6: 20%	–
57	18	30	2.93	Right Inferior Frontal Gyrus (pars Opercularis)	Area 44: 50%	Area 44
63	9	21	2.81	Right Precentral Gyrus	Area 6: 20%;	–
					Area 44: 20%	
42	12	30	2.32	Right Inferior Frontal Gyrus (pars Opercularis)	Area 45: 10%	–
36	12	−27	2.77	Right Temporal Pole	–	–
−6	15	36	2.58	Left Middle Cingulate Cortex	–	–
57	−15	−6	2.31	Right Superior Temporal Gyus	–	–

The corresponding brain region and the probability and assignment to a probabilistic-anatomical map is displayed as calculated with the SPM Anatomy Toolbox [14]. Three peaks in left insular cortex (*) were activated by listening to actively learned melodies in contrast to listening to passively learned melodies at the following statistical threshold: p<0.005, K>100, Z-scores>3.0. Table 1 also shows additional peaks activated by the same contrast at a lower statistical threshold: p<0.05, K>50, Z-scores>2.25.

There were no significant effects at the high threshold (p<0.005, k>100, Z>3.9) in the correlation analyses with ratings of tendency to movement imagination, number of learning trials, and familiarity differences between the two classes of melodies. At the lower threshold (p<0.05, Z>2.25, k>50), results from the correlation analyses with the subjects' ratings of their tendency to movement imagination are summarized in Tab. 2. Areas with a positive correlation to imagination ratings included the primary visual, primary somatosensory, and primary motor area. Positive regression with the number of learning trials needed to reach the learning criterion showed predominantly sub-cortical effects, especially in the cerebellum (Tab. 3), while negative correlations with the number of learning trials were observed pre-dominantly in cerebral cortical areas, in particular in the dorsal premotor cortex (Tab. 4). Finally, a correlation with familiarity differences between actively and passively learned melodies was, among other areas, found in Broca's area (BA 45, Tab. 5). Importantly, even at the low statistical threshold, none of the correlation analyses showed effects in the anterior insular cortex.

**Table 2:** Summary of peak locations of BOLD signal change correlated to the subjects' tendency to movement imagination (p<0.05, K>50, Z-scores>2.25).

MNI-Coordinates (x/y/z)			Z-Scores	Brain region	Probability	Assigned to
39	−66	27	2.88	Right Middle Occipital Gyrus	–	–
−9	−54	27	2.6	Left Precuneus	–	–
24	−42	−6	2.48	Right Parahippocampal Gyrus	–	–
30	−27	−6	2.33	Right Hippocampus	Hippocampus (FD): 10%;	–
					Hippocampus (CA): 10%	
−21	−39	48	2.31	Left Somatosensory Cortex	Area 3a: 40%;	Area 3a
					Area 3b: 10%	
−36	12	−30	3.7	Left Temporal Pole	–	–
−54	6	−27	2.77	Left Middle Temporal Gyrus	–	–
−30	−96	18	3.17	Left Occipital Cortex	–	–
−21	−93	−6	2.44	Left Occipital Cortex	Area 18: 30%;	–
					Area 17: 20%	
−45	−69	21	2.39	Left Middle Temporal Gyrus	–	–
−39	−75	39	2.28	Left Middle Occipital Gyrus	–	–
27	3	−33	2.74	Right Parahippocampal Gyrus	Hippocampus (EC): 70%	Hippocampus (EC)
54	3	−30	2.63	Right Middle Temporal Gyrus	–	–
−27	−45	−12	2.67	Left Furiform Gyrus	–	–
24	−93	6	2.66	Right Middle Occipital Gyrus	Area 17: 50%	Area 17
33	−33	48	2.64	Right Postcentral Gyrus	Area 3a: 50%	Area 3a
−51	−15	33	2.59	Left Postcentral Gyrus	Area 3b: 50%	Area 3b
−45	−6	33	2.5	Left Precentral Gyrus	Area 4p: 30%	Area 4p
−15	51	36	2.41	Left Superior Frontral Gyrus	–	–
−57	−21	−9	2.27	Left Middle Temporal Gyrus	–	–
−48	−24	−15	2.27	Left Middle Temporal Gyrus	–	–
−60	−36	0	2.26	Left Middle Temporal Gyrus	–	–

Conventions like in table 1.

**Table 3:** Summary of peak locations of BOLD signal change positively correlated to the number of learning trials the subjects needed before reaching the learning criteria (p<0.05, K>50, Z-scores>2.25).

MNI-Coordinates (x/y/z)			Z-Scores	Brain region	Probability	Assigned to
18	−9	−9	2.76	Right Medial Temporal Lobe	Hippocampus: 10%; Amygdala: 10%	–
−15	12	−6	2.51	Left Putamen	–	–
24	−27	−27	2.38	Right Cerebellum	–	–
−30	−84	−33	3.15	Left Cerebellum	–	–
−15	−69	−51	2.5	Left Cerebellum	–	–
−42	−69	−36	2.48	Left Cerebellum	–	–
−21	54	0	2.75	Left Superior Frontal Gyrus	–	–
24	−81	−42	2.75	Right Cerebellum	–	–
33	−60	−51	2.54	Right Cerebellum	–	–
−18	−33	−24	2.5	Left Cerebellum	–	–
33	33	−6	2.37	Right Inferior Frontal Gyrus (pars orbitalis)	–	–

Conventions as in table 1.

**Table 4:** Summary of peak locations of BOLD signal change negatively correlated to the number of learning trials the subjects needed before reaching the learning criteria (p<0.05, K>50, Z-scores>2.25).

MNI-Coordinates (x/y/z)			Z-Scores	Brain region	Probability	Assigned to
30	−51	75	2.72	Right Superior Parietal Lobule	–	–
42	18	−27	3.1	Right Temporal Pole	–	–
51	18	−18	2.57	Right Temporal Pole	–	–
−12	−21	54	2.88	Left Dorsal Premotor Cortex	Area 6: 40%	Area 6
−9	−6	42	2.88	Left Middle Cingulate Cortex	Area 6: 10%	–
0	21	18	2.81	Anterior Cingulate Cortex	–	–
−39	12	36	2.78	Left Middle Frontal Gyrus	Area 44: 20%	
−42	−6	3	2.75	Left Rolandic Operculum	–	–
−45	−57	42	2.73	Left Inferior Parietal Lobule	hIP1: 20%	–
−45	−27	27	2.25	Left Parietal Operculum	OP 1: 20%	–
30	−15	51	2.54	Right Dorsal Premotor Cortex	Area 6: 60%	Area 6
60	15	27	2.39	Right Frontal Gyrus (pars opercularis)	Area 45: 40%	–
45	3	45	2.27	Right Precentral Gyrus	Area 6: 20%	–
−42	42	9	2.36	Left Inferior Frontal Gyrus (pars tringularis)	Area 45: 10%	–
30	−51	75	2.72	Right Superior Parietal Lobule	–	–

Conventions as in table 1.

**Table 5:** Summary of peak locations of BOLD signal change positively correlated with individual familiarity-differences (familiarity with actively learned minus passively learned melodies, p<0.05, K>50, Z-scores>2.25).

MNI-Coordinates (x/y/z)			Z-Scores	Brain region	Probability	Assigned to
33	−72	48	3.43	Right Superior Parietal Lobule	–	–
36	−69	36	2.89	Right Middle Occipital Gyrus	–	–
57	33	12	3.16	Right Frontal Gyrus (pars triangularis)	Area 45: 80%	Area 45
51	36	30	3.09	Right Middle Frontal Gyrus	–	–
−36	−66	48	2.72	Left Inferior Parietal Lobule	–	–
−18	3	57	2.52	Left Superior Frontal Gyrus	Area 6: 20%	–
−18	−15	51	2.29	Left Superior Frontal Gyrus	Area 6: 30%	–
36	−36	45	2.46	Right Postcentral Gyrus	Area 2: 90%	Area 2
24	9	69	2.31	Right Superior Frontal Gyrus	–	–

Conventions like in table 1.

**Figure 4:**
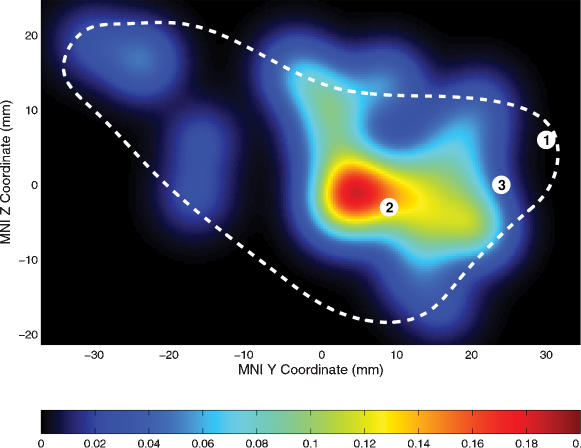
Relation of response peaks to the insular cortex sensorimotor hand area. The likely position of the hand area of the insular cortex was determined by a meta-analysis or previous fMRI studies [16]–[35]. Activation likelihood estimates (ALEs, see Methods section for further details) are color coded. The highest likelihood for hand movement-related activation was in a region from approx. Y = 0 to Y = 20 and from Z = −10 to Z = 10. The peak locations of the present study are indicated by white discs; numbers refer to the rows of Tab. 1. Peak 2 was located in the region with high ALE. The dashed line indicates the approximate outline of the left insula.

The activation likelihood estimate (ALE) map obtained from the meta-analysis of hand movement-related fMRI responses in the insular cortex is shown in Fig. 4. Highest ALE values were found in the region of the anterior insular cortex ranging from approx. Y = 0 to Y = 20 and Z = −10 to Z = 10. This region included activation found in the actively learned>passively learned contrast in our study (peak ‘2’ in Fig. 4).

## Discussion

In the present study we show a change in sound-elicited brain activations when the same sounds are associated with active hand movement, and that this effect is evident already after a short (30 minutes and less) time of training. To investigate sound-action association effects, we used a paradigm in which subjects learned to play two simple melodies on the piano. Brain responses associated with listening to these ‘actively learned melodies’ were compared with those associated with ‘passively learned melodies’, that is, melodies with which subjects were familiarized by passive listening only. We found significantly increased fMRI responses to the actively learned melodies in the left anterior insular cortex, overlapping the insular sensorimotor hand representation area as determined by meta-analysis of published hand movement-related insular fMRI responses [16]–[35].

Our finding of a singular activation site stands in contrast to the extended temporo-parieto-premotor circuit that has been described in a recent fMRI study [1] as activated during listening to everyday manual action-related sounds. But at a lower statistical threshold we found effects in a more widespread temporo-frontal network that included the right middle temporal gyrus, as also found by Gazzola and colleagues [1]. The differences between the cortical network in our vs. Gazzola et al's study (e.g. the involvement of posterior parietal areas in the latter) might in part be due to different responses to simple everyday action sounds, such as ripping a sheet of paper, than to sounds associated with finger movement sequences such as those required for playing the piano melodies in our study. Additionally, the sound-action associations investigated by Gazzola et al. [1] were established over long periods – months to years - before the experiment. The activation of the anterior insula observed in our study may therefore represent an early stage of auditory-motor learning, which may be later consolidated into a different network more similar to the one identified by Gazzola and co-workers [1].

The idea that long-term training for the paradigm used in our study would induce other changes than those seen after a single, short training session is also supported by two studies that have used similar melody learning paradigms and have tracked the ensuing brain response differences over multiple training sessions [3], [5]. In the following we will however mainly focus the discussion on the effects that were reported after the first training session.

Bangert and colleagues [3] have investigated cortical activation patterns using DC-EEG-recordings obtained in subjects who passively listened to a musical piece before and after subjects learned to play the melody on the piano with their right hand. The scalp topography of slow DC-potential changes evoked by passively listening to the musical piece was recorded from 30 electrode positions. Two groups were investigated: one using a piano with a conventional key-to-pitch assignment (as in our study), and one group with a random assignment. Differences in slow EEG potentials were found before and after the first training session that were particularly wide-spread in the subject group with conventional key-to-pitch assignment. How do these results compare with the fMRI activations that we observed in the present study? With regard to the anterior insular activation, it is difficult to predict whether and how neuronal activity in this region would show up in scalp surface EEG recordings. Generally, there is good evidence that deep cortical sources can contribute to the scalp EEG (cf. the cingulate motor area sources [37]). Source reconstruction results also suggest the principal existence of significant insular contributions to the EEG recorded from the surface of the scalp [38], [39]. Although the exact generator sites of the scalp potentials reported by Bangert and co-workers [3] can not be determined by visual inspection of the reported data, it is in view of the preceding considerations possible that some of the EEG changes observed in this study might have originated in the insular cortex. The wide-spread topography of the reported EEG changes, together with the multiple frontal and temporal fMRI activation sites we have obtained in our study at a low statistical threshold, indicate that a rather widespread cortical network is additionally activated by listening to a rehearsed as compared with an unrehearsed piece. Further investigations will be needed to delineate this network and the function of its nodes more thoroughly.

In a second study employing a melody learning paradigm, D'Ausilio and colleagues [5] evaluated motor cortical excitability using TMS during passive listening to piano melodies that were previously rehearsed compared with control melodies. They found increased motor cortical excitability for the rehearsed but not for the unrehearsed pieces. Specifically, they found increased intracortical facilitation (ICF) after the first learning session, and increased ICF and motor evoked potentials (MEPs) after long-term training. These findings give additional support to the view that auditory-motor co-activations might be induced by a single short training session. However, one may ask how these TMS findings may be reconciled with the fact that neither the fMRI results of our study nor those of Gazzola and co-workers [1] showed primary motor cortex activation when subjects listened to action-related sounds. Interestingly, similar discrepancies also exist for everyday manual action sounds that elicited TMS effects [40] but no primary motor fMRI effects [1] and also for action observation in the visual domain: visual observation of natural hand movements modulates MEPs recorded from hand muscles [41]. An involvement of the primary motor cortex (M1) in visual action observation was also indicated by source reconstruction of magneto-encephalographic data [42]. In contrast, no M1 activation was found in several PET [43] and fMRI [44]–[46] studies on visual action observation. An explanation for these differences could be sensitivity differences between the methods used, i. e. weak functional effects might already be evident when applying TMS but not show up in fMRI. In this context it is also interesting to note that in our study primary sensorimotor cortex activation, but not anterior insular cortex activation, was correlated with the tendency of the subjects to imagine hand movement during listening to the actively learned melodies. Therefore, between-study differences in the subjects' tendency to movement imagination may be an additional cause for whether or not primary motor cortex involvement is found in different studies.

In addition to movement imagination, another potential confounding factor for the type of study we have performed are differences in familiarity between the actively and passively learned melodies. In the studies by Bangert et al. [3] and D'Ausilio et al. [5] the subjects' familiarity with the different melodies used as stimuli was not evaluated. In the present study, we have assessed familiarity using a melody-variation detection task (Fig. 2). A correlation analysis with the individual familiarity differences between actively and passively learned melodies showed correlations in multiple frontal and parietal regions, in particular in Broca's area (BA 45). Importantly, even at low statistical threshold, no correlation was observed in the insular cortex. The same was true for additional correlations with the number of learning trials needed by the subjects to reach the learning criterion and with the subjects' tendency to movement imagination. The primary visual cortex activation correlation found in the later case might be related to the fact that subjects' ratings regarding the mode of movement imagination were predominantly visual. Our results lend therefore little support to the possibility that familiarity differences, speed of learning, or movement imagination made a major contribution to the increased fMRI responses that we found in the insular cortex.

These responses in the insula overlapped with the insular sensorimotor hand area as determined by a meta-analysis of previous functional imaging studies [16]–[35]. It is thus possible that the re-activation of movement representations acquired during the preceding training session may be a mechanism underlying the effects we have observed in the anterior insula, i. e. that these effects represent a ‘mirror property’ of the insular cortex. The left anterior insula contains a somatotopic motor map that includes representations of finger, shoulder, and leg movement [47], and has a role in speech production [48]. Furthermore, the anterior insula has been found to be active during imagery of standing and walking [49], and has been reported to be involved in the ‘sense of agency’ of hand movement [50], that is, the experience of oneself being the cause of an action, which is a fundamental aspect of action representation. Additionally, hand-movement related regional cerebral blood flow changes have also been found in the fronto-opercular cortex [13]. Whether our findings have any relation to the auditory ‘mirror neurons’ as described in macaque premotor cortex that respond to both action execution and to listening to the sounds related to the same action [51] remains to be determined.

The present study is a first step toward delineating the exact brain areas involved in short-time auditory-motor learning. Several perspectives for further investigations on auditory-motor learning arise from the present study: One would be to repeat the present experiments with the left hand, or a similar experiment with different body parts, in order to determine lateralization and somatotopy of the ensuing effects. Such experiments could provide more evidence for the postulated relation to learned movement representations. A further perspective is to use functional imaging for investigating the neuronal basis of auditory-motor learning over longer periods of time and in relation to that of classical motor learning, where specific changes occur on time scales ranging from minutes to years [52], [53].
